# Surgical Management of Adult-Onset Artery From the Pulmonary Artery (ALCAPA): A Narrative Review of Surgical Techniques

**DOI:** 10.7759/cureus.104488

**Published:** 2026-03-01

**Authors:** Chandler Pugh, Kristina Snoddy, Erin Reid, Adam Witcher

**Affiliations:** 1 Medicine, Edward Via College of Osteopathic Medicine, Auburn, USA; 2 Medicine, UAB St. Vincent's East, Birmingham, USA; 3 Cardiothoracic Surgery, UAB St. Vincent's East, Birmingham, USA

**Keywords:** anomalous origin of the left coronary artery from pulmonary artery (alcapa), coronary artery bypass grafting(cabg), coronary reimplanation, takeuchi, takeuchi repair

## Abstract

Anomalous left coronary artery from the pulmonary artery (ALCAPA) is a rare congenital coronary anomaly in which the left coronary artery originates from the pulmonary artery, resulting in myocardial ischemia due to coronary steal after postnatal reduction in pulmonary artery pressure. Most patients present in infancy with heart failure, but a subset survives into later childhood or adulthood through extensive collateralization from the right coronary artery. Late presenters typically demonstrate a dilated, tortuous right coronary artery with retrograde flow into the pulmonary artery. Surgical correction is recommended at all ages; however, optimal management in older patients remains debated. This review summarizes the existing literature and discusses surgical strategies for adult-onset ALCAPA.

Surgical repair of adult-onset ALCAPA aims to eliminate coronary steal and restore a physiologic two-coronary system. The choice of technique depends on anatomic factors, including the origin of the left coronary artery, its distance from the aorta, vessel length, and the extent of intercoronary collaterals. Direct reimplantation of the left coronary artery into the aorta is preferred when feasible, as it provides durable restoration of normal coronary physiology, though it may be limited by tension or inadequate coronary length. The Takeuchi procedure offers an alternative when reimplantation is not possible but carries risks such as baffle stenosis and pulmonary artery obstruction. Coronary artery bypass grafting may be considered in selected adults, though competitive flow from collaterals and long-term graft durability remain concerns. Additionally, new techniques such as modified Cabrol and modified Takeuchi may offer even greater promise for the future but will require further research to establish their place in the field.

## Introduction and background

Anomalous left coronary artery from the pulmonary artery (ALCAPA), also known as Bland-White-Garland syndrome, is a rare congenital anomaly presenting in approximately 1 in 300,000 live births and accounts for 0.25-0.5% of congenital heart defects [1,2]. The abnormal origin of a coronary artery from the pulmonary artery was first reported by Brooks in 1882; however, a comprehensive clinical description of the condition was not published until 1933 by Bland, White, and Garland, who described the syndrome in association with cardiac hypertrophy [3,4]. ALCAPA is most commonly an isolated cardiac defect. Still, it can occur in combination with atrial septal defect, ventricular septal defect, patent ductus arteriosus, tetralogy of Fallot, aorto-pulmonary window, and coarctation of the aorta [5]. The pathophysiology of ALCAPA likely begins embryonically, with abnormal separation of the conotruncus into the aorta and pulmonary artery (PA) or from persistence of the pulmonary buds and involution of the aortic buds, which eventually form the coronary arteries [6].

While ALCAPA most commonly presents in infants, approximately 10-15% of patients survive into adulthood due to well-developed collateral circulation from the right coronary artery (RCA) to the left coronary artery (LCA) [7]. This robust collateralization prevents the typical infantile presentation, though these patients remain at risk for myocardial ischemia, left ventricular dysfunction, mitral regurgitation, and ventricular arrhythmias [6]. Both infant-type and adult-type presentations are characterized by ischemic cardiomyopathy due to inadequate left ventricular perfusion; however, the clinical presentation varies widely across age groups [6,8].

In infants, ischemia is typically acute, with symptoms including crying during feeding, failure to thrive, tachypnea, cyanosis, fatigue, and colic [8,9]. In the neonatal period, when pulmonary artery pressure remains elevated near systemic levels, the anomalous left coronary artery receives relatively well-oxygenated blood at adequate pressure from the pulmonary artery, transiently protecting against severe ischemia. As pulmonary vascular resistance falls during the first weeks of life, pulmonary artery pressure decreases to approximately one-half of systemic pressure within 24 hours and continues to decline to one-third or less by 2 weeks of age. The LCA now receives only deoxygenated blood at low pressure from the pulmonary artery, which is insufficient for adequate myocardial perfusion [10]. Collateral vessels develop from the right coronary artery to supply the left ventricular territory, but because pulmonary artery pressure is lower than left ventricular pressure, blood flows retrograde from the left ventricular myocardium through these collaterals and drains into the low-pressure pulmonary artery, resulting in coronary steal [8,9,11]. This creates a left-to-right shunt at the coronary level, diverting blood away from myocardial perfusion and causing severe left ventricular ischemia, myocardial infarction, and heart failure, precipitating acute decompensation in infancy [12].

In adults, survival is established by robust collateralization from the RCA, but coronary steal persists, and cardiomyopathy is thus chronic. Patients often present with dyspnea, angina, palpitations, arrhythmias, syncope, and rarely sudden cardiac death, rather than acute systolic heart failure in infants [13]. Cardiomegaly, mitral regurgitation, and vastly dilated collateral arteries are often present [14]. Among adults, the average age of presentation is 41, but it is highly variable. Patients who are more physically active may present earlier, but ALCAPA can present as late as 75 years of age [15,16].

This paper will focus on the adult presentation of ALCAPA and will serve to examine surgical techniques for adult ALCAPA patients. Direct reimplantation, the Takeuchi procedure, coronary artery bypass grafting (CABG) with left internal mammary artery (LIMA) ligation, and the modified Cabrol technique will be reviewed. Among these, direct reimplantation is the most frequently performed and generally preferred approach, followed by the Takeuchi and CABG approaches. 

## Review

Pathophysiology and diagnosis

In normal anatomy, the LCA arises from the aortic trunk superiorly to the left cusp of the aortic valve [17]. However, in ALCAPA, the LCA arises from the PA rather than the aortic arch and can have several possible origins [18]. Most commonly, the LCA arises from the left-facing sinus of the main pulmonary artery (MPA), or more rarely from the right-facing or non-facing sinus [19-21]. While origin from the pulmonary sinus is the most common pathology in around half of cases, the LCA can also arise from the MPA (41.66% of cases) and the right pulmonary artery (8.3% of cases) [22]. The origin of the LCA does not usually alter symptomatology; rather, it plays an important role in determining the surgical approach, which will be discussed later.

**Figure 1:**
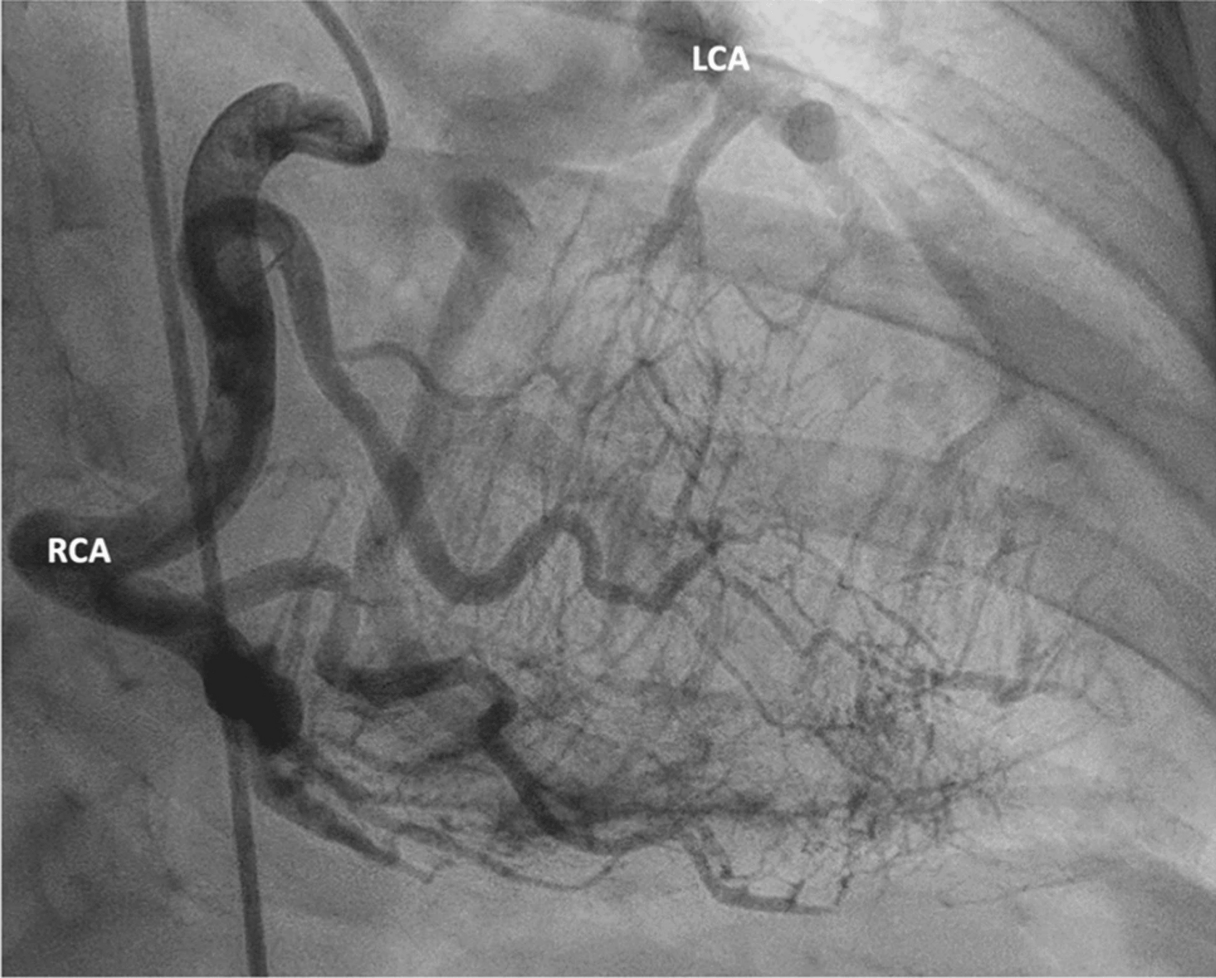
Angiogram of an adult patient with ALCAPA. Angiogram demonstrating the typical findings of angiography in adult patients with ALCAPA, a dilated and tortuous right coronary artery with intercoronary collaterals. There is retrograde filling of the left coronary artery via collateral branches from the right coronary artery, and the left coronary artery originates from the main pulmonary artery [24]. Reproduced from Stolz et al., Clinical Research in Cardiology (2025), distributed under the Creative Commons Attribution 4.0 International (CC BY 4.0) license.

Adult ALCAPA is almost universally characterized by a dilated and tortuous RCA with well-developed intercoronary collaterals, features that are not present in the infantile form [16,20]. A dilated and tortuous RCA is shown in Figure 1. Despite the development of robust collateral vessels, RCA-derived flow cannot provide adequate long-term perfusion due to progressive coronary steal into the low-pressure PA. Once demand exceeds the capacity of the collaterals, symptoms will emerge [23]. Many patients thus present with an acute myocardial infarction due to progressive coronary steal and undergo an invasive coronary evaluation, which reveals the diagnosis of ALCAPA [16]. However, ALCAPA can also be diagnosed incidentally in patients who undergo a transthoracic echocardiogram (TTE) or electrocardiogram (ECG) for unrelated reasons [6].

**Figure 2:**
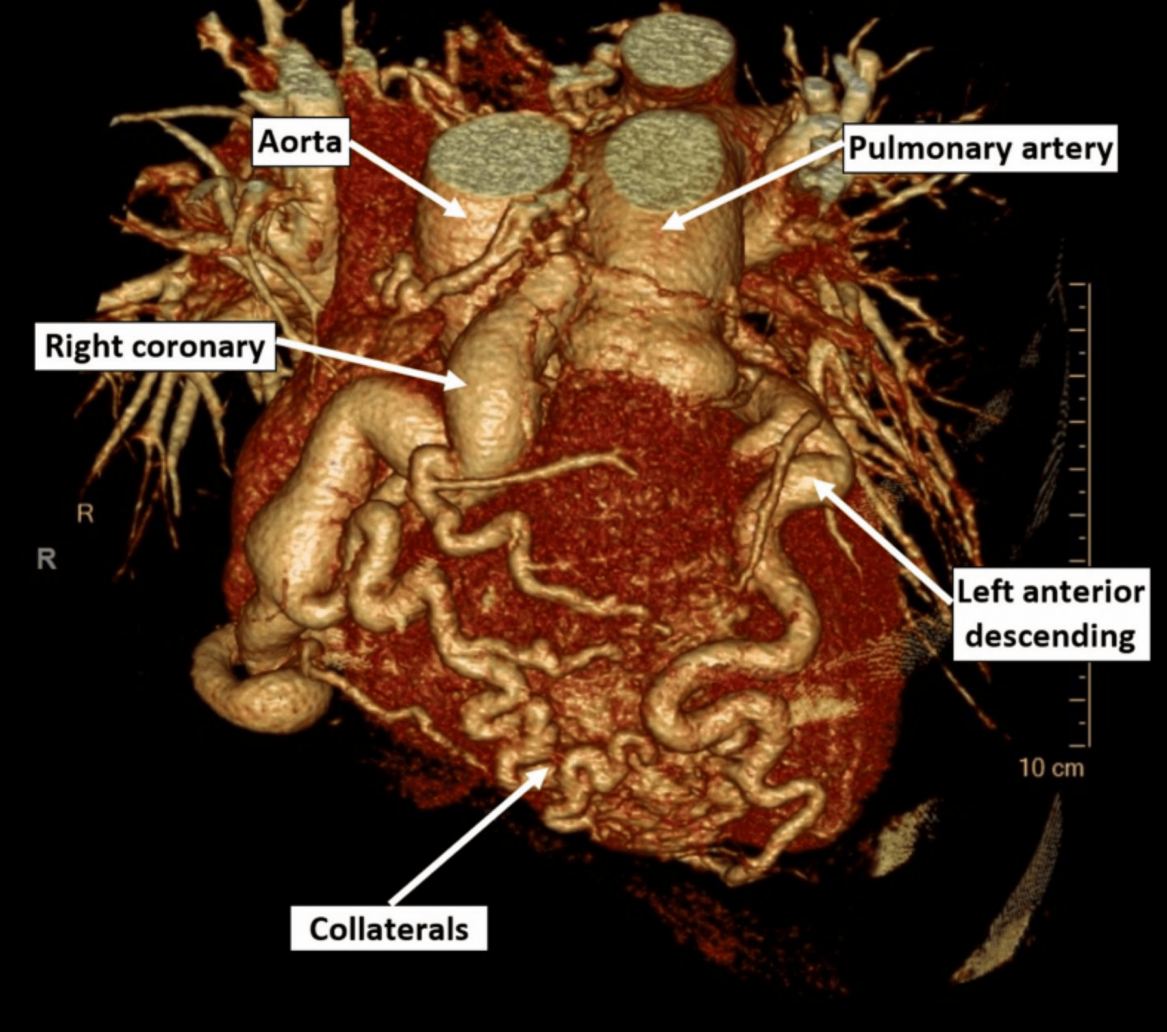
Cardiac CTA of a patient with ALCAPA. Cardiac CTA demonstrates collaterals from the right coronary artery to the left ventricle [29]. Reproduced from Liu et al., CJC Open (2023), distributed under the Creative Commons Attribution (CC BY) license.

**Figure 3:**
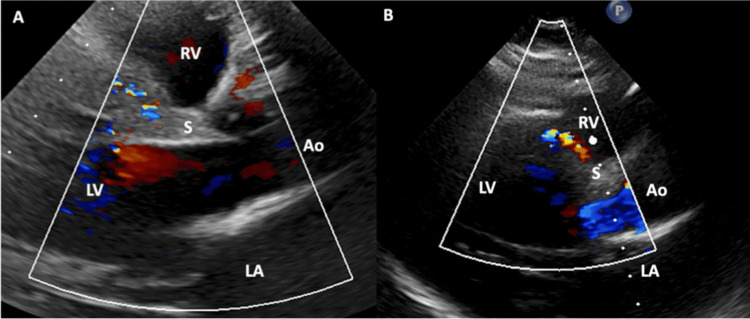
Echocardiography with Color Doppler in a patient with ALCAPA. A: Abnormal septal TTE color Doppler signal due to an excessive septal collateral system seen in ALCAPA [24]. B: Abnormal septal TTE color Doppler signal due to an excessive septal collateral system seen in ALCAPA [24]. Ao: Aorta; LA: Left Atrium; LV: Left Ventricle; RV: Right Ventricle; S: Interventricular Septum Reproduced from Stolz et al., Clinical Research in Cardiology (2025), distributed under the Creative Commons Attribution 4.0 International (CC BY 4.0) license.

Per the 2025 American Heart Association Guidelines, coronary angiography, computed tomography angiography (CTA), and cardiac magnetic resonance imaging (CMRI) are the recommended methods for evaluating an anomalous coronary artery in adults to demonstrate a dilated and tortuous RCA. CTA is generally preferred for superior resolution (Figure 2), although CMR may provide adequate imaging of the PA and pulmonary sinus. Coronary angiography by catheterization can be helpful when there is concern about coronary artery stenosis [25]. TTE and ECG, while not recommended by official guidelines, can be diagnostic and are non-invasive. Diagnostic findings on TTE (Figure 3) include retrograde blood flow from the LCA to the PA and a dilated, tortuous RCA. Intracoronary collaterals may be demonstrated on color Doppler [26]. Other findings may include mitral regurgitation, dilated collateral coronary arteries, ventricular dysfunction, and increased echogenicity of the papillary muscles [27]. ECG findings include Q waves, ST depressions, and T-wave changes in the anterolateral leads I, AVL, V5, and V6 [28].

The treatment approach for patients with ALCAPA, whether discovered acutely or incidentally, is surgery [25]. However, in older adults, particularly those who are asymptomatic or those with low-risk anatomy, such as in the absence of stenosis, a conservative approach may be considered due to the increased risks associated with surgery [16,25].

Methods

A narrative literature review was performed using PubMed, MEDLINE, and Google Scholar databases. Search terms included combinations of “ALCAPA,” “anomalous left coronary artery,” “adult ALCAPA,” “late-presenting ALCAPA,” “Takeuchi repair,” “coronary reimplantation,” “coronary artery bypass grafting,” and “Cabrol technique.” Only English-language publications were included.

Eligible studies consisted of adult and adolescent case reports, case series, retrospective cohort studies, and systematic reviews describing surgical management strategies, perioperative outcomes, and long-term follow-up. Pediatric-only cohorts were excluded unless they provided comparative insight relevant to adult surgical decision-making.

Extracted variables included patient age at diagnosis, presenting symptoms, coronary anatomy and site of anomalous origin, imaging modalities utilized, operative technique employed, postoperative complications, and survival or functional outcomes. Given the rarity of late-presenting ALCAPA and the heterogeneity of available data, formal meta-analysis was not feasible. Findings were therefore synthesized qualitatively, with emphasis on reproducible surgical principles and outcome trends.

Surgical techniques

Direct Reimplantation

**Figure 4:**
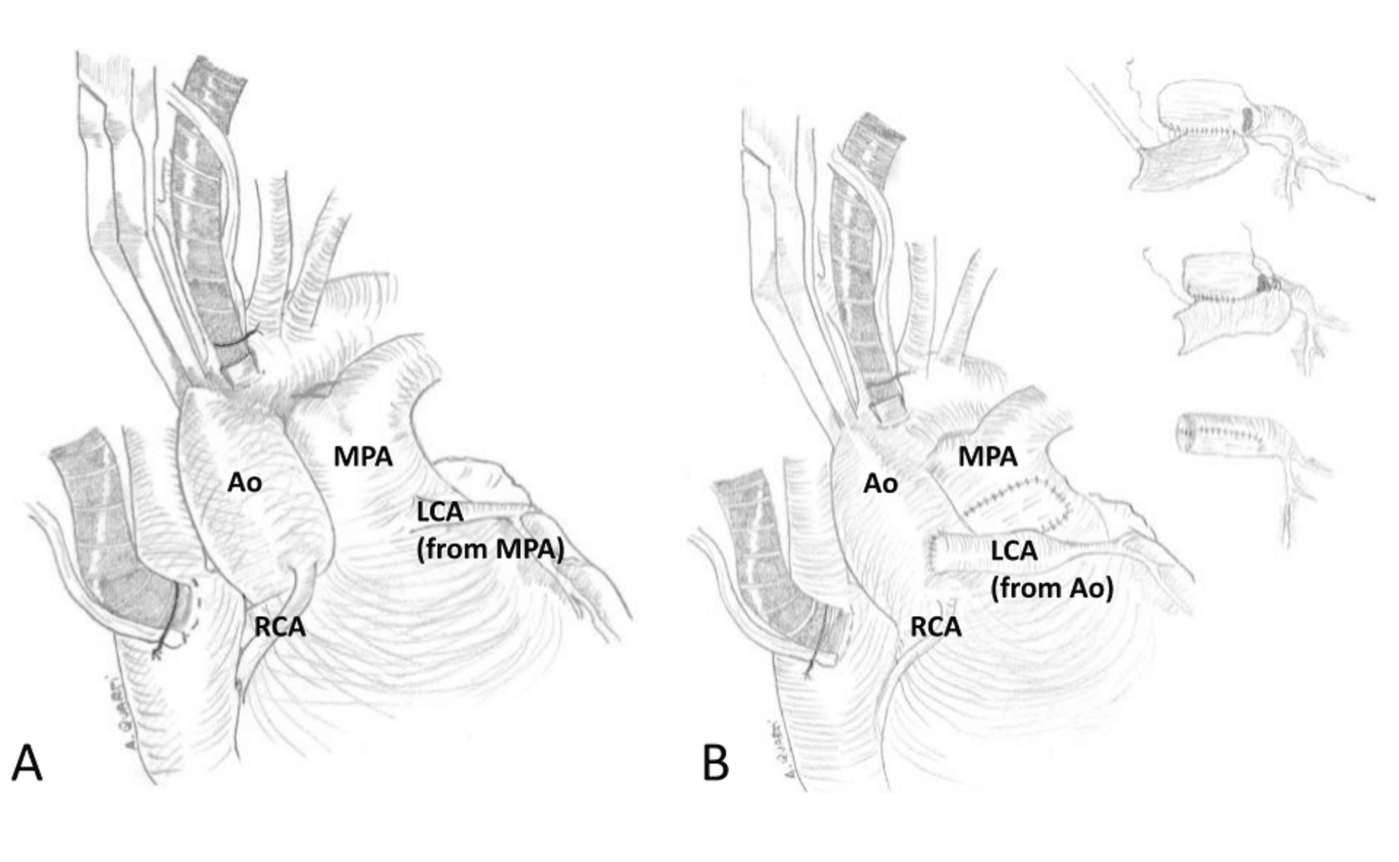
Diagram of direct coronary reimplantation in a patient with ALCAPA. A: Anatomy present in a patient with ALCAPA [31]. B: The creation and relocation of the conduit (see right) as part of direct coronary reimplantation [31]. Ao: Aorta; LCA: Left Coronary Artery; MPA: Main Pulmonary Artery; RCA: Right Coronary Artery Adapted from Quarti et al., Case Reports in Medicine (2009), distributed under the Creative Commons Attribution (CC BY) license.

Direct reimplantation of the anomalous LCA from the PA into the aorta reestablishes the normal two-coronary system, as demonstrated in Figure 4. The ascending aorta and MPA are dissected free from surrounding tissues to ensure full mobilization and avoid injury to the LCA. The MPA is transected, and the LCA is carefully detached from the pulmonary root with a generous cuff of surrounding arterial wall tissue to preserve the integrity of the ostium. The proximal LCA is mobilized toward its bifurcation to ensure adequate length for tension-free reimplantation. A neo-ostium is then created in the left coronary sinus of the aorta. The LCA is reimplanted end-to-end into the neo-ostium using a running 6-0 polypropylene suture, beginning at the deepest point and proceeding bilaterally to complete the anastomosis. The defect in the PA root is reconstructed with a non-treated autologous pericardial patch using a continuous 6-0 polypropylene suture, ensuring restoration of pulmonary root structure. The repair is completed with continuous closure of the pulmonary patch margins, ensuring hemostasis and restoration of normal great vessel alignment [30].

Limitations of the direct reimplantation technique include feasibility based on anatomy. Direct reimplantation is limited in stenotic or fibrosed LCAs, which are often unmanipulable [20]. In other scenarios, the LCA may originate from a site difficult to access during the procedure, such as a distal location on the PA, from the lateral wall of the PA, or the non-facing pulmonary sinus. Performing direct reimplantation when the LCA arises from these locations can result in excessive tension or distortion due to the larger distance between the anomalous LCA and the aorta [32-34]. In such cases, coronary artery elongation techniques can be utilized with direct reimplantation, or other methods can be considered [35,36].

Takeuchi Method

Dr. Takeuchi et al. in 1979 established an innovative approach to establishing a dual-coronary system without direct coronary translocation by creating an intrapulmonary tunnel that redirects oxygenated blood from the aorta to the anomalous LCA, thereby restoring myocardial perfusion. This technique is a suitable alternative when direct reimplantation of the LCA onto the aorta is not feasible due to anatomical constraints [37]. A transverse incision is made just below the bifurcation of the PA to determine the origin of the LCA accurately. Following aortic cross-clamping, antegrade cardioplegia is administered while manually compressing the LCA ostium to ensure optimal myocardial protection. A box-shaped incision is then fashioned in the anterior wall of the MPA. Simultaneously, an inverse “L”-shaped aortotomy is performed. The PA flap and the adjacent aortic wall are then excised in a face-to-face manner to facilitate the creation of an aortopulmonary window. This connection establishes a direct pathway for oxygenated blood from the aorta to reach the intrapulmonary tunnel. The PA flap is then positioned to fully enclose the LCA orifice, with special care to extend coverage beyond the posterior commissure. The anterior wall of the PA is subsequently reconstructed using an autologous pericardial patch. This refined version of the procedure reflects technical advances and long-term refinements since Takeuchi's original description, while preserving the core principle of rerouting aortic blood into the anomalous LCA [32].

The Takeuchi technique avoids direct manipulation of the LCA to avoid kinking or excess stress on the LCA and restores normal coronary circulation via an intrapulmonary baffle [38,39]. The technique avoids direct manipulation, allowing its use in older patients with less vessel mobility and in those with a narrowed (but not occluded) LCA [39,40]. Limitations of the Takeuchi technique include PA stenosis around the baffle, baffle leaks or obstructions, and a high reoperation rate due to baffle leaks and PA stenosis [39,41-43].

However, modifications to the Takeuchi technique have been pioneered to reduce the risk of baffle leaks, chiefly by altering the shape or material of the baffle or reinforcing the tunnel. Such procedures are known as the modified Takeuchi method [44]. The baffle can be adapted with synthetic materials such as Gore-Tex or autologous pericardial patches to reduce leaks and improve outcomes [41,45].

Coronary Artery Bypass Grafting (CABG)

Coronary artery bypass grafting should be considered in patients without a patent LCA, as in those with coronary artery disease, or when the anatomy is not suitable for either direct reimplantation or the Takeuchi procedure [46,47]. CABG is performed via ligation of the abnormal LCA from the pulmonary artery with coronary revascularization using a LIMA graft to the LAD.

Early attempts at surgical correction of ALCAPA included the use of saphenous vein grafts to restore coronary perfusion. In 1977, eight patients received saphenous vein grafts, which remained patent for the 8-year monitoring period [48]. Similar positive findings have been replicated with other studies demonstrating resolution of angina for 2-8 years [48-50]. The benefits include feasibility when anatomy is not suitable for direct repair, a good early safety profile, restoration of dual coronary circulation, and improved long-term graft patency when arterial conduits are used compared to venous conduits [27,46,49]. The risks include perioperative mortality due to massive infarcts, long-term graft failure, which is increased with venous grafts, and an overall limited scope of evidence. There is also concern for the maturation of an arterial graft to the distally bilateral vessel with major collaterals from the RCA and the long-term patency of vein grafts in a young population [19]. Additionally, redo surgeries and complex reoperations carry an increased risk [51]. While CABG can be effective in select ALCAPA cases, its use should be tailored to patient-specific factors, with careful weighing of risks and benefits to ensure the best possible outcomes.

Modified Cabrol Technique

The modified Cabrol technique has been reported in a case study where anatomical constraints prevented direct reimplantation. Therefore, a polytetrafluoroethylene (PTFE) graft was chosen to reduce anatomical stress caused by distance and vessel orientation. PTFE is a more rigid material than traditional Dacron, which can decrease the likelihood of graft kinking when the vessel length is inadequate. Modified Cabrol was successful on a one-year follow-up and was thus deemed successful [19]. More research will likely be needed to determine its place in the future of ALCAPA repairs; however, it has considerable potential.

Heart Transplant

An orthotopic heart transplant has not been extensively performed in ALCAPA. In a case study, a 46-year-old woman with symptomatic New York Heart Association (NYHA) functional classification Class IV heart failure underwent orthotopic heart transplant. She returned to full functional status [52]. Thus, a heart transplant can be useful in ALCAPA when irreversible myocardial damage has occurred, assuming there are donors available. Further research would be needed to validate this case study, but it does hold promise for a rare diagnosis and presentation.

Surgical decision making in the patient with ALCAPA

**Figure 5:**
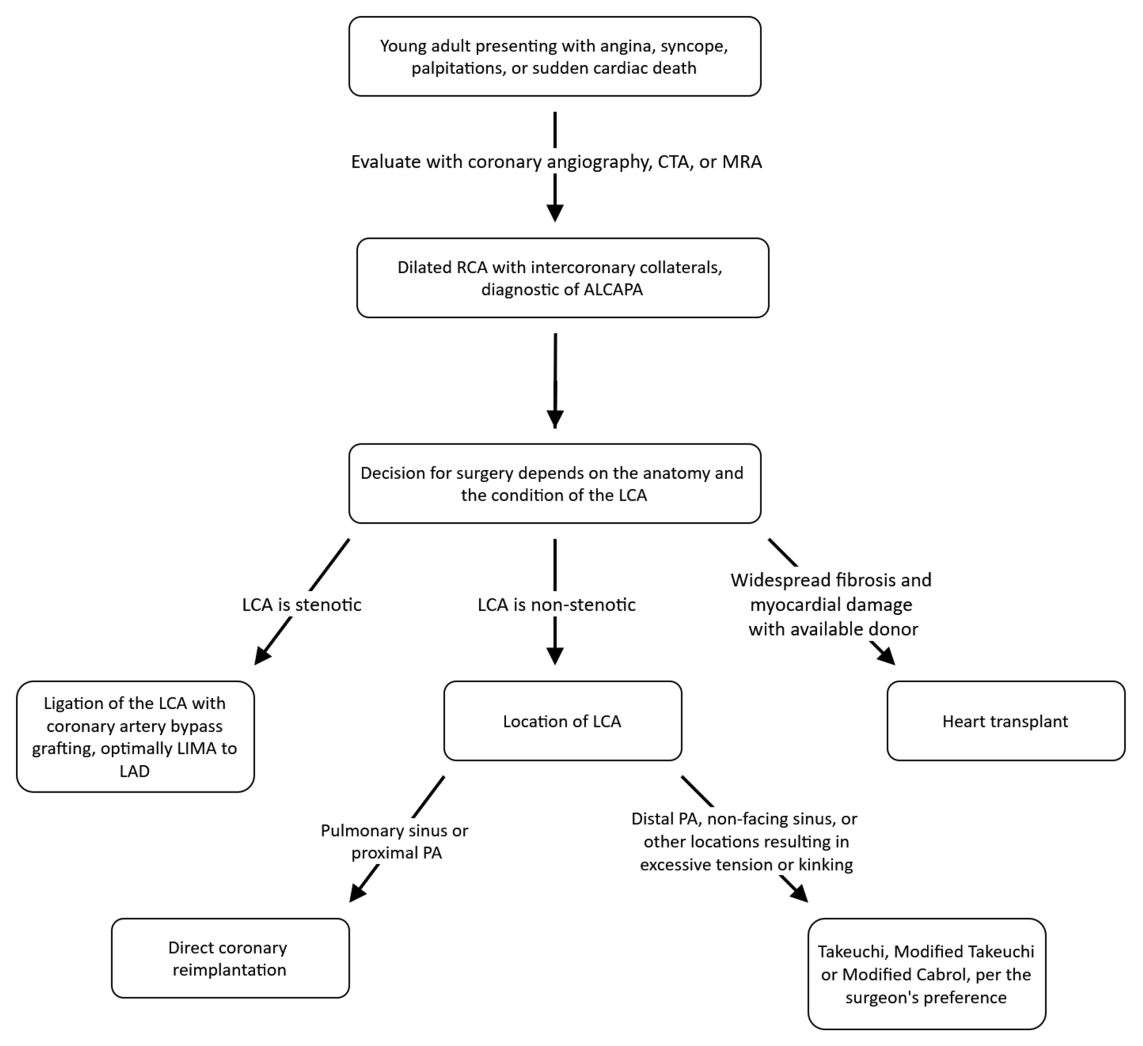
Our suggested algorithm for the surgical approach to a patient with ALCAPA (anomalous left coronary artery from the pulmonary artery). CTA: Computed Tomography Angiography; MRA: Magnetic Resonance Angiography; RCA: Right Coronary Artery; ALCAPA: Anomalous Left Coronary Artery from the Pulmonary Artery; LCA: Left Coronary Artery; LIMA: Left Internal Mammary Artery; LAD: Left Anterior Descending (Artery); PA: (Pulmonary Artery)

When approaching the patient with ALCAPA, the decision on which surgical technique to use should primarily be based on the anatomy, as shown in our suggested algorithm in Figure 5.

If the LCA originates from the pulmonary sinus or from the proximal PA, direct coronary reimplantation is recommended, as this technique restores normal coronary anatomy and physiology [31,53]. However, origins from the non-facing pulmonary sinus or the lateral wall of the PA are likely to be more technically challenging due to the larger distance between the anomalous LCA and the aorta in adult patients. In such cases, direct reimplantation remains preferred, with coronary artery elongation techniques used as needed [35,36].

If the LCA is more distal on the PA, direct reimplantation can result in excessive tension or distortion of the LCA; thus, in such cases, the Takeuchi technique should be considered [32-34]. If the LCA is fibrosed or stenotic, CABG is preferred, as the LCA is unlikely to be amenable to manipulation, and bypassing around the abnormal vessel is preferred [20]. If the patient has widespread fibrosis with NYHA Class IV heart failure and a donor is available, transplantation can be considered, though evidence is extremely limited [52]. Conservative therapy can be considered in an elderly, asymptomatic patient with favorable anatomy [16,25].

## Conclusions

Despite the rarity of adults presenting with ALCAPA, its surgical management requires excessive surgical planning and consideration. Various vessel patterning and general myocardial viability should be considered when planning the intervention strategy. Additionally, early intervention is essential, especially in young patients, as untreated ALCAPA may result in sudden cardiac death. The surgical management of adult-onset ALCAPA remains a complex case-by-case challenge, but clinical guidelines have become clearer with time.

Direct reimplantation remains the gold standard when anatomically suitable. However, when anatomical limitations exist, various alternative procedures should be considered, including the Takeuchi method, the modified Takeuchi method, the modified Cabrol technique, CABG, and heart transplant. Regarding both the modified Takeuchi method and the modified Cabrol technique, further research will be necessary to assess their validity and value in adult ALCAPA repair. Tailored surgical strategies at specialized centers offer the best outcomes with close follow-up. As collective experience grows, procedural selection and clinical guidelines will continue to improve. However, further research is needed to evaluate options beyond direct reimplantation, especially in patients with anatomical limitations.
